# Knowledge Management Orientation: An Innovative Perspective to Hospital Management

**Published:** 2017-12

**Authors:** Matina GHASEMI, Mazyar GHADIRI NEJAD, Kemal BAGZIBAGLI

**Affiliations:** 1. Faculty of Tourism, Eastern Mediterranean University, TRNC, Famagusta, Turkey; 2. Young Researchers and Elite Club, South Tehran Branch, Islamic Azad University, Tehran, Iran; 3. Dept. of Economics, Faculty of Business and Economics, Eastern Mediterranean University, TRNC, Famagusta, Turkey

**Keywords:** Knowledge management area, Innovation, Hospital management

## Abstract

**Background:**

By considering innovation as a new project in hospitals, all the project management’s standard steps should be followed in execution. This study investigated the validation of a new set of measures in terms of providing a procedure for knowledge management-oriented innovation that enriches the hospital management system.

**Methods:**

The relation between innovation and all the knowledge management areas, as the main constructs of project management, was illustrated by referring to project management standard steps and previous studies. Through consultations and meetings with a committee of professional project managers, a questionnaire was developed to measure ten knowledge management areas in hospital’s innovation process. Additionally, a group of experts from hospital managers were invited to comment on the applicability of the questionnaires by considering if the items are measurable in hospitals practically.

**Results:**

A close-ended, Likert-type scale items, consisted of ten sections, were developed based on project management body of knowledge thorough Delphi technique. It enables the managers to evaluate hospitals’ situation to be aware whether the organization follows the knowledge management standards in innovation process or not. By pilot study, confirmatory factor analysis and exploratory factor analysis were conducted to ensure the validity and reliability of the measurement items.

**Conclusion:**

The developed items seem to have a potential to help hospital managers and subsequently delivering new products/services successfully based on the standard procedures in their organization. In all innovation processes, the knowledge management areas and their standard steps help hospital managers by a new tool as questionnaire format.

## Introduction

Revolution in hospital management requires new procedures and a paradigm shift in today’s highly competitive world. Hospitals will be more successful by documenting any systematic and well-organized procedure. This concern is critical for hospital managers around the world without regard to nationality (1). Improvement in hospital management system is considered by emphasizing more on hospitals’ system (2) and consequently on successful hospital management and competitive strategies (3). The competitive strategy in hospital management has changed in different ways by focusing more on their managerial skills (4, 5) and necessity of professional project managers (Professional project managers in this study were certified from the Project Management Institute where is a US nonprofit professional organization for project management. (6).

Hospitals always have challenges of both the external and internal environments (7). The only way for managers to overcome these challenges is improving their managerial skills (5, 8). For instance, one of the main challenges in hospitals is medical tourism. Patients travel to overseas destinations with the aim of receiving medical treatments (9). Medical tourism is a unique industry, which is a combination of both medical, and tourism industry in a specified location (10). This competitive area highlights the importance of being innovative and systematic in the quest of attracting more medical tourists (11). The hospitals, particularly as the main organization in this framework should follow some standard procedures that could bolster the competitiveness of the medical tourism market (12).

The procedures, undertaken in any hospital, are critical for its competitiveness and performance (13). In previous studies in hospital management, just one or two knowledge management areas were considered like procurement management (14); cost management (15, 16) and communication management (17, 18). There is no study until yet which consider all ten knowledge management areas in a comprehensive analysis in hospitals; especially regard innovation processes.

Innovation is a priority for managers in hospital management (19). Any new project has its procedures and standards to deliver the final product or service successfully. In project management, any new idea should follow standard steps in different categories. These categories are the ten knowledge management areas which are; integration, scope, time, cost, quality, human resource, communication, risk, procurement, and stakeholder management according to project management body of knowledge (PMBOK) (6). As stated earlier (20), projects are the lifeblood of every organization and project management is better, faster and cheaper way for success. By referring to scholars’ opinions, which hinted the relationship between each knowledge management area and innovation, the main purpose of current study is aware managers about the importance of professional analysis by using the developed items as a new tool in questionnaire format. Before, there were not any measurement items, which comprehensively measure new projects’ standard steps in hospitals (Table 1).

## Methods

Project management in hospitals is an area that has not yet been fully explored in innovation processes. A qualitative research method was adopted in this study to illustrate the importance of knowledge management areas and innovation by delivering a new tool for these main constructs’ measurement. The development and validation of the measure items occurred in the following sections:

### The standard steps of PMBOK

According to PMBOK (6) as any novel idea is performing for the first time, should be defined as a new project. In the current study, any innovation in hospitals is considered as a new project too. According to this standard, projects firstly need to be undertaken by all ten knowledge management areas to be successful in execution (Table 1).

**Table 1:** Project management processes

***Knowledge Management Areas***	***Project Management Process Groups***				
	***Initiating***	***Planning***	***Executing***	***Monitor and Controlling***	***Closing***
Integration Management	Develop Project Charter	Develop Project Management Plan	Direct and Manage Project Work	Monitor and Control Project Work Perform Integrated Change Control	Close Project or Phase
Scope Management		Plan Scope Management. Collect Requirements Define Scope Create WBS		Validate Scope Control Scope	
Time Management		Plan Schedule Management. Define Activities Sequence Activities Estimate Activity Resources Estimate Activity Durations Develop Schedule		Control Schedule	
Cost Management		Plan Cost Management Estimate Costs Determine Budget		Control Costs	
Quality Management		Plan Quality Management.	Perform Quality Assurance	Control Quality	
Human Resource Management		Plan Human Resource Management	Acquire Project Team Develop Project Team. Manage Project Team		
Communication Management		Plan Communications Management	Manage Communication	Control Communications	
Risk Management				Control Risks	
		Plan Risk Management. Identify Risks Perform Qualitative Risk Analysis Perform Quantitative Risk Analysis Plan Risk Responses			
Procurement Management		Plan Procurement Management	Conduct Procurements	Control Procurements	Close Procurement
Stakeholder Management	Identify Stakeholders	Plan Stakeholder Management	Manage Stakeholder Engagement	Control Stakeholder Engagement	

### Delphi technique

By applying Delphi technique, a committee of eight professional project managers from Iran was asked to participate in developing the ten new close-ended, Likert-type scale questionnaires. All PMBOK standard procedures (Table 1) should be applied in any industry, but as all of them were converted to questionnaires format, then it was important to analyze it with the committee by the following question: 1) if the steps could be applied to hospitals? 2) if the steps need any changes according to the hospitals’ systems?

The committee members carefully considered the suitability of the questionnaires, especially for hospital purposes, and were asked 3) whether different functional managers could answer them or not. Items identification and scale development are described in the results section. Using the items according to PMBOK, the committee drafted a preliminary version of the measurement items.

The procedures and instructions pointed out by (21–23) which illustrated all the steps regarding how to develop questionnaires were considered in detail for the current study purpose.

In the next step, to verify the clarity and applicability of the questionnaires, an expert review was conducted by the ten participating intending hospital managers after designing the questionnaires. The managers were selected regardless if they are from international or local hospitals. The expert review aimed to assess if the items in each measured item were relevant and whether the selected items adequately represented the ten knowledge management areas.

## Results

The developed questions will help hospital managers to measure each knowledge management area for any novel idea, which will be called knowledge management- oriented innovation questionnaires. By using these questionnaires, managers will be sure about the innovation’s success in the hospitals. Likert-type scale (1 = strongly disagree; 5 =strongly agree) response categories were used for the following items which were drawn from Table 1. A pilot study was conducted on 40 questionnaires. Questions were answered by targeting participants from international hospitals’ general managers and functional managers like a quality manager, risk manager and human resource manager for example. Respondents were assured that there were no wrong or right answers to the items in the questionnaires. These items ( Appendix 1) were answered just by managerial levels as the items deal with professional analysis regard innovation. The language of the questionnaire is English, and official language in Iran is Persian then back translation has been performed for the current study. Confirmatory factor analysis (CFA) and exploratory factor analysis (EFA) were considered to ensure the reliability and validity of the measurement items (Appendix 1).

## Discussion

This study seeks to expand our understanding of how the method of analyzing knowledge management areas could be helpful in the innovation process that improves hospital management processes. The result delivers a new tool in a questionnaire format that helps hospital managers to follow a standard procedure regarding innovation. The items have been used to measure the effect of knowledge management-oriented innovation.

### Importance of knowledge management areas and innovation

Innovation is highly relevant in today’s’ competitive environment (24). Innovation could be defined as the only way to convert an opportunity to bring benefits earnable by an organization (25). The strategic approach of knowledge management is a key factor for innovation in both theory and practice (26). In a professional organization that is familiar with knowledge management, any novel idea that is implemented for the first time should be defined as a project because it is and requires all the standard steps of project management to deliver a successful new product or service (6). For using the standard steps of all knowledge management areas in innovation, it is necessary to demonstrate the relationship, separately as follow: Integration management and innovation (27, 28); Scope management and innovation (29); Time management and innovation (30); Cost management and innovation (31); Quality management and innovation (32); Human Resource management and innovation (33, 34); Communication management and innovation (35, 36); Risk management and innovation(37); Procurement management and innovation (38) and, Stakeholder management and innovation (39).

### Hospital Management and Knowledge Management Areas

The importance of knowledge management areas in hospitals is pointed out by many scholars. For instance human resource management (40, 41); stakeholder management (42–44); procurement management (14); cost management (15, 16) and communication management (17, 18).

Only some knowledge management areas were examined in hospitals management studies, which were designed to represent their importance like human resource management (40) and stakeholder management (42).

A novel idea is not subjected to a comprehensive analysis (45) which increases the probability of these ideas’ failure. As processes are critical to the success of hospital systems (1), the current study will be helpful for hospital managers to deliver new products and services to patients.

## Conclusion

Current study delivers a new measurement tool by emphasizing the importance of all the ten knowledge management areas in hospital management and by introducing them as the main categories in the innovation process.

For future studies, researchers are suggested to determine which knowledge management area has more significant effect on innovation in hospitals by using a quantitative research approach. The lack of professional project managers in hospitals could be one of the main causes of failure of innovation. Therefore, a qualitative research approach could be conducted in hospitals on knowledge management areas regard innovation to discover the general knowledge of managers.

## Ethical considerations

Ethical issues (Including plagiarism, informed consent, misconduct, data fabrication and/or falsification, double publication and/or submission, redundancy, etc.) have been completely observed by the authors.

## Appendix 1:

**Table:**

**Integration Management-oriented Innovation**
In our hospital, a charter is developed for each new idea to demonstrate all the details.
In our hospital, a plan is developed for each new idea.
In our hospital, managers for each new idea direct and manage the project.
In our hospital, managers for each new idea monitor and control the project.
In our hospital, managers for each new idea perform integrated change control.
In our hospital, each new idea will be closed and classified after being delivered.
**Scope Management-oriented Innovation**
In our hospital, managers for each new idea have a scope management plan.
In our hospital, managers for each new idea collect all the requirements.
In our hospital, managers for each new idea define the scope of the work.
In our hospital, managers for each new idea create a work breakdown structure.
In our hospital, managers for each new idea validate the scope of the work.
In our hospital, managers for each new idea control the scope of the work.
**Time Management-oriented Innovation**
In our hospital, managers for each new idea have a schedule management plan.
In our hospital, managers for each new idea define the activities.
In our hospital, managers for each new idea arrange the activities in sequence.
In our hospital, managers for each new idea estimate the activity resources.
In our hospital, managers for each new idea estimate the activity duration.
In our hospital, managers for each new idea develop a schedule.
In our hospital, managers for each new idea control the schedule.
**Cost Management-oriented Innovation**
In our hospital, managers for each new idea have a cost management plan.
In our hospital, managers for each new idea estimate the costs.
In our hospital, managers for each new idea determine the budget.
In our hospital, managers for each new idea control the related costs.
**Quality Management-oriented Innovation**
In our hospital, managers for each new idea have a quality management plan.
In our hospital, managers for each new idea perform quality assurance.
In our hospital, managers for each new idea control the quality.
**Human Resource Management-oriented Innovation**
In our hospital, managers for each new idea have a human resource management plan.
In our hospital, managers for each new idea acquire a project team.
In our hospital, managers for each new idea develop the project team.
In our hospital, managers for each new idea manage the project team.
**Communication Management-oriented Innovation**
In our hospital, managers for each new idea have a communication management plan.
In our hospital, managers for each new idea manage communication.
In our hospital, managers for each new idea control communication.
**Risk Management-oriented Innovation**
In our hospital, managers for each new idea have a risk management plan.
In our hospital, managers for each new idea identify the risks.
In our hospital, managers for each new idea perform a qualitative risk analysis.
In our hospital, managers for each new idea perform a quantitative risk analysis.
In our hospital, managers for each new idea have a risk response plan.
In our hospital, managers for each new idea control the risks.
**Procurement Management-oriented Innovation**
In our hospital, managers for each new idea have a procurement management plan.
In our hospital, managers for each new idea conduct the procurements by contracts.
In our hospital, managers for each new idea control all the procurement contracts.
In our hospital, managers for each new idea close all the procurement contracts.
**Stakeholder Management-oriented Innovation**
In our hospital, managers for each new idea identify all the stakeholders.
In our hospital, managers for each new idea have a stakeholder management plan.
In our hospital, managers for each new idea manage stakeholder engagement.
In our hospital, managers for each new idea control stakeholder engagement.

## References

[1] Griffith JR White KR (2005). The revolution in hospital management. J Healthc Manag, 50(3):170–89. 15974333

[2] Pink GH McKillop I Schraa EG Preyra C Montgomery C Baker GR (2001). Creating a balanced scorecard for a hospital system. J Health Care Finance, 27(3):1–20. 14680029

[3] Cleverley WO Harvey RK (1992). Competitive strategy for successful hospital management. Hosp Health Serv Adm, 37(1):53–69. 10116113

[4] Kebede S Abebe Y Wolde M Bekele B Mantopoulos J Bradley EH (2010). Educating leaders in hospital management: a new model in Sub-Saharan Africa. Int J Qual Health Care, 22(1):39–43. 19951963 10.1093/intqhc/mzp051 PMC2803009

[5] Supic ZT Bjegovic V Marinkovic J Milicevic MS Vasic V (2010). Hospital management training and improvement in managerial skills: Serbian experience. Health Policy, 96(1):80–9. 20116126 10.1016/j.healthpol.2010.01.002

[6] Project Management Institute (2013). A Guide to the Project Management Body of Knowledge (PMBOK® Guide). 5th ed Project Management Institute, Newtown Square, Pa.

[7] Rechel B Dubois CA McKee (2006). The health care workforce in Europe: learning from experience. World health organization.

[8] Gregory D Baigelman W Wilson IB (2003). Hospital economics of the hospitalist. Health Serv Res, 38(3):905–918. 12822918 10.1111/1475-6773.00152 PMC1360922

[9] Lunt N Horsfall D Hanefeld J (2016). Medical tourism: A snapshot of evidence on treatment abroad. Maturitas, 88:37–44. 27105695 10.1016/j.maturitas.2016.03.001

[10] Cannon Hunter W (2007). Medical Tourism: A New Global Niche. Int J Tourism Sci, 7(1):129–40.

[11] Saadatnia F Mehregan MR (2014). Determining and Prioritizing Factors Affecting Customers Attraction of Medical Tourism from the Perspective of Arabic Countries (Case Study: Iran-Mashhad Razavi Hospital). Int J Marketing Stud, 6(3):155.

[12] Hjalager AM (2009). Innovations in travel medicine and the progress of tourism—Selected narratives. Technovation, 29(9):596–601.

[13] Cleven A Mettler T Rohner P Winter R (2016). Healthcare quality innovation and performance through process orientation: Evidence from general hospitals in Switzerland. Technol Forecast Soc Change, 113:386–95.

[14] Ginsburg G (2005). Human factors engineering: A tool for medical device evaluation in hospital procurement decision-making. J Biomed Inform, 38(3):213–9. 15896694 10.1016/j.jbi.2004.11.008

[15] Wu VY (2009). Managed care’s price bargaining with hospitals. J Health Econ, 28(2):350–60. 19108922 10.1016/j.jhealeco.2008.11.001

[16] Culler SD Jevsevar DS McGuire KJ Shea KG Little KM Schlosser MJ (2017). Predicting the Incremental Hospital Cost of Adverse Events Among Medicare Beneficiaries in the Comprehensive Joint Replacement Program During Fiscal Year 2014. J Arthroplasty, 32(6):1732–8. 28185753 10.1016/j.arth.2017.01.003

[17] Sharpe B Hemsley B (2016). Improving nurse–patient communication with patients with communication impairments: hospital nurses’ views on the feasibility of using mobile communication technologies. Appl Nurs Res, 30:228–36. 27091283 10.1016/j.apnr.2015.11.012

[18] Berger ZD Boss EF Beach MC (2017). Communication behaviors and patient autonomy in hospital care: A qualitative study. Patient Educ Couns, 100(8):1473–81. 28302341 10.1016/j.pec.2017.03.006

[19] Miller FA French M (2016). Organizing the entrepreneurial hospital: Hybridizing the logics of healthcare and innovation. Research Policy, 45(8):1534–44.

[20] Kodukula P (2014). Organizational Project Portfolio Management: A Practitioner’s Guide. ed. J. Ross publishing.

[21] Oppenheim AN (1968). Questionnaire design and attitude measurement. ed. Heinemann, London.

[22] Crano WD Brewer MB Lac A (2014). Principles and methods of social research. Routledge.

[23] Foddy W (1993). Constructing Questions for Interviews and Questionnaires: Theory and Practice in Social Research. ed. Cambridge University Press, Cambridge.

[24] Novelli M Schmitz B Spencer T (2006). Networks, clusters and innovation in tourism: A UK experience. Tourism Management, 27(6):1141–52.

[25] Huse M Neubaum D Gabrielsson J (2005). Corporate Innovation and Competitive Environment. The International Entrepreneurship and Management Journal, 1(3):313–33.

[26] Valkokari K Paasi J Rantala T (2012). Managing knowledge within networked innovation. Knowledge Management Research and Practice, 10(1):27–40.

[27] Pavlou PA El Sawy OA (2011). Understanding the Elusive Black Box of Dynamic Capabilities. Decision Sciences, 42(1):239–73.

[28] Salazar MR Lant TK Fiore SM Salas E (2012). Facilitating Innovation in Diverse Science Teams Through Integrative Capacity. Small Group Res, 43(5):527–58.

[29] Saleh F Ryan C (1992). Client perceptions of hotels: a multi-attribute approach. Tourism Management, 13(2):163–8.

[30] Peters PF (2006). Time, innovation and mobilities: travel in technological cultures. Taylor & Francis.

[31] Martĺnez-Ros E Orfila-Sintes F (2009). Innovation activity in the hotel industry. Technovation, 29(9):632–41.

[32] Cohen WM Levinthal DA (1990). Absorptive Capacity: A New Perspective on Learning and Innovation. Administrative Science Quarterly, 128–52.

[33] Seibert SE Kraimer ML Crant JM (2001). What do proactive people do? A longitudinal model linking proactive personality and career success. Personnel Psychology, 54(4):845–74.

[34] Bruns V Holland DV Shepherd DA Wiklund J (2008). The Role of Human Capital in Loan Officers’ Decision Policies. Entrepreneurship Theory and Practice, 32(3):485–506.

[35] Homburg C Kuehnl C (2014). Is the more always better? A comparative study of internal and external integration practices in new product and new service development. J Bus Res, 67(7):1360–7.

[36] Lui SS (2009). The Roles of Competence Trust, Formal Contract, and Time Horizon in Interorganizational Learning. Organization Studies, 30(4):333–53.

[37] Gurd B Helliar C (2017). Looking for leaders: ‘Balancing’ innovation, risk and management control systems. The British Accounting Review, 49(1):91–102.

[38] Haugbølle K Pihl D Gottlieb SC (2015). Competitive Dialogue: Driving Innovation Through Procurement? Procedia Economics and Finance, 21:555–62.

[39] Ommen NO Blut M Backhaus C Woisetschläger DM (2016). Toward a better understanding of stakeholder participation in the service innovation process: More than one path to success. J Bus Res, 69(7):2409–16.

[40] Jończyk JA (2015). The Impact of Human Resource Management on the Innovativeness of Public Hospitals in Poland. Procedia-Social and Behavioral Sciences, 213:1000–7.

[41] Qingwei F (2012). Research on Evaluation Index System of Management Effectiveness on Hospital Human Resource Based on Balanced Scorecard. Procedia Environ Sci, 12:1040–4.

[42] Zehir C Çınar F Şengül H (2016). Role of Stakeholder Participation between Transparency and Qualitative and Quantitative Performance Relations: An Application at Hospital Managements. Procedia-Social and Behavioral Sciences, 229:234–45.

[43] Bravi F Gibertoni D Marcon A Sicotte C Minvielle E Rucci P Angelastro A Carradori T Fantini MP (2013). Hospital network performance: A survey of hospital stakeholders’ perspectives. Health Policy, 109(2):150–7. 23201189 10.1016/j.healthpol.2012.11.003

[44] Buchanan D Jordan S Preston D Smith A (1997). Doctor in the process. The engagement of clinical directors in hospital management. J Manag Med, 11(2–3):132–56. 10173243 10.1108/02689239710177774

[45] Jenson I Leith P Doyle R West J Miles MP (2016). Innovation system problems: Causal configurations of innovation failure. J Bus Res, 69(11), 5408–12.

